# Co-design of the neurodevelopment assessment scale

**DOI:** 10.3389/frcha.2025.1497632

**Published:** 2025-01-28

**Authors:** Tsz Ying Wong, Syeda Ishra Azim, Christa Lam-Cassettari, Ping-I. Lin, Antonio Mendoza Diaz, Alicia Montgomery, Anne Masi, Kylie-Ann Mallitt, Andrew Whitehouse, Mark R. Dadds, Valsamma Eapen

**Affiliations:** ^1^ School of Clinical Medicine, Discipline of Psychiatry and Mental Health, UNSW Medicine & Health, The University of New South Wales, Sydney, NSW, Australia; ^2^ Department of Psychiatry and Behavioral Neuroscience, Saint Louis University School of Medicine, St. Louis, MO, United States; ^3^ Tasmanian Centre for Mental Health Service Innovation, Tasmanian Health Service, Hobart, TAS, Australia; ^4^ School of Psychology, The University of Tasmania, Hobart, TAS, Australia; ^5^ Sydney School of Public Health, Faculty of Medicine and Health, The University of Sydney, Sydney, NSW, Australia; ^6^ Telethon Kids Institute, The University of Western Australia, Perth, WA, Australia; ^7^ School of Psychology, The University of Sydney, Sydney, NSW, Australia

**Keywords:** neurodevelopmental disorder, transdiagnostic assessment, co-design, consumer and community involvement, psychological assessment

## Abstract

**Introduction:**

Neurodevelopmental disorders (NDDs) have high comorbidity rates and shared etiology. Nevertheless, NDD assessment is diagnosis-driven and focuses on symptom profiles of individual disorders, which hinders diagnosis and treatment. There is also no evidence-based, standardized transdiagnostic approach currently available to provide a full clinical picture of individuals with NDDs. The pressing need for transdiagnostic assessment led to the development of the Neurodevelopment Assessment Scale (NAS).

**Methods:**

This paper describes the co-design process used in the development of NAS prototype with stakeholders including individuals with NDDs, parents of children with NDDs, and health professionals.

**Results and discussion:**

Results indicated stakeholder consensus that NAS would be useful for NDD assessment, and included recommendations for fine-tuning the way some questions were asked (e.g., child's diagnoses), question flow (e.g., branching logic), and the language and presentation of the prototype (e.g., readability). Stakeholders also suggested the administration protocol should be flexible using electronic, face-to-face, online formats etc.

## 1 Introduction

Neurodevelopmental disorders (NDDs) are a heterogeneous group of conditions which include cerebral palsy (CP), intellectual disability (ID), autism spectrum disorder (ASD), attention-deficit/hyperactive disorder (ADHD), tic disorders, as well as motor, communication and speciﬁc learning disorders (1, 2). These disorders share risk-conferring genetic variants (3–7), as well as environmental risk factors (8, 9). Furthermore, NDDs often share similar symptoms, for example, sleeping issues (e.g., 10), executive function (e.g., 11, 12), attention (e.g., 13, 14), and social information processing discrepancies (e.g., 15). The term “Early Symptomatic Syndromes Eliciting Neurodevelopmental Clinical Examinations” (ESSENCE) has been proposed, highlighting the fact individuals with NDDs share common symptoms (16).

The close relation between NDDs is also evidenced by the high co-occurrence rate of two or more NDD diagnoses (i.e., comorbidity) (17–21);. For example, Eapen and Robertson (22) found that 86.5% of individuals with Tourette syndrome had comorbidities such as ADHD. Similarly, a review found that Tourette syndrome frequently co-occurs with ADHD (17%–68% of the cohort with Tourette syndrome also had ADHD) (23). Furthermore, ADHD also frequently co-occurs with ASD, learning disorders, and tic disorders (24), while ASD also frequently co-occur with ADHD, ID, and developmental coordination disorder (DCD) (25). The close relation between NDDs has led to the hypothesis that NDDs are a continuous spectrum of disorders (26, 27).

Despite the close relationship between NDDs, NDDs have been traditionally categorized as individual disorders (1). The diagnostic process of NDDs is often conducted in silos (28). Assessment tools, procedures, and diagnostic criteria for NDDs are also often designed to diagnose a specific NDD (29, 30). This approach is prone to the omission of different aspects of clinical proﬁles (11, 12). As a result, the diagnosis of comorbidities could be delayed (31, 32). Furthermore, treatment of NDDs could be compromised by misdiagnoses caused by overlapping symptoms across NDDs, and a failure to address associated symptoms and dysfunctions (2, 33). In the broader sense of knowledge advancement, siloed diagnostic approaches limit understanding of NDDs as a whole, as they do not disentangle the complex presentation of individual NDDs and the interface with comorbidities (5, 27).

To reduce the compartmentalization of NDD diagnoses which creates an illusion that NDDs are mutually exclusive, a comprehensive and transdiagnostic tool should be developed. We report on the co-design process used to develop an evidence-based and standardized transdiagnostic Neurodevelopment Assessment Scale (NAS; protocol as described by Masi and colleagues) (34). NAS is designed to be used across NDD symptoms, broader comorbidity symptoms, associated distress, and family history, in a single assessment.

NAS was co-designed with stakeholders to ensure the needs and requirements of future end-users (individuals with NDDs, parents/careers, and health care professionals) were considered and incorporated into NAS. Co-design refers to designing and deriving a solution to a question together with stakeholders (35). Compared to the traditional researcher-orientated designing process, co-design enhances the understanding of the problem, and provides additional perspectives in the development of the solution (36). For NAS development, stakeholders provided feedback through survey items with options as well as qualitative feedback as part of the co-design process. This paper summarizes stakeholder feedback on the NAS prototype (second consultation as described below).

## 2 Materials and methods

The ethics approval of this study was obtained from the UNSW Human Research Ethics Committee (reference number: HC210260).

### 2.1 Participants/stakeholders

Stakeholders were recruited via promotion through practitioner networks, disability service providers, advocacy groups, and word-of-mouth. Participation was voluntary, and participants could withdraw anytime without consequences. Stakeholders were given an information sheet and provided consent if they agreed to participate. Eligibility criteria included: (a) being 18 years old or over; and (b) being a parent/caregiver of a child with an NDD, an adult with an NDD, a health professional or clinician working with children with NDDs, or a representative of a disability service provider. Participants were ineligible to participate if they were non-English speakers, although no consenting stakeholders were excluded on this basis.

The initial pool (see “Design”) of stakeholder participants consisted of nine health practitioners/disability service providers, seven parents of children with NDDs, as well as seven adults with NDDs (i.e., *n* = 23). Participants were invited to introduce themselves, and amongst adults with NDDs, NDDs they reported having included: ASD (*n* = 5), ADHD (*n* = 4), CP (*n* = 1), dyslexia (*n* = 1), developmental coordination disorder (*n* = 1), and NDDs that were not further elaborated (*n* = 1). Other diagnoses included: anxiety symptoms (*n* = 2), obsessive-compulsive disorder (*n* = 1), brain injury (*n* = 1), and unspecified mental health issues (*n* = 1). In terms of parents and caregivers, three participants reported that they were mothers of children with NDDs, two reported that they were fathers, and two did not specify their relationship with their children with NDDs. The type of NDDs that they reported their children having included: ASD (*n* = 6), tic disorders (*n* = 2), ADHD (*n* = 1), ID (*n* = 2), learning difficulties (*n* = 1), and stuttering (*n* = 1). Other conditions that parents reported their children having included premature birth (*n* = 2), obsessive-compulsive disorder (*n* = 1), anxiety symptoms (*n* = 1), bipolar disorder (*n* = 1), and pontocerebellar hypoplasia (*n* = 1). Regarding health professionals, clinicians, and representatives of disability service providers, they reported that they were: pediatricians (*n* = 4), speech therapists (*n* = 2), social worker (*n* = 1), disability physiotherapist (*n* = 1), and unspecified clinician (*n* = 1). Although all health professionals reported that they work with individuals with NDDs, most did not specify the type of NDDs (*n* = 5), and examples given included ID (*n* = 2), ADHD (*n* = 1), ASD (*n* = 1), and learning disorders (*n* = 1).

The second consultation included six stakeholders within each respective participant group [three health practitioners/disability service providers, one parent, and one adult with NDD(s) withdrew from participation], yielding a total of 18 stakeholders. Due to the anonymous nature of the study, it was not possible to find out the demographics of the participants who dropped out using data from the first consultation. Stakeholders were given AUD20 worth of supermarket vouchers as a token of appreciation.

### 2.2 Design

The study involved a mixed-methods co-design process. A cluster analysis of archived data sets, which contained assessment results of individuals with NDDs, was conducted to investigate how symptoms of different NDDs could be categorized. The identified categories of symptoms were used to guide the structure of NAS. In addition, stakeholders provided anonymous comments via questionnaires, and comments were aggregated to inform the formation (first consultation) and modification (second consultation) of the first NAS prototype. The first consultation explored stakeholders' thoughts on what was important in the process of assessing NDDs, as well as stakeholders' preferences on NDD assessments through open-ended questions. Details of the second stakeholder consultation are described in the “Second Stakeholder Consultation” section below. This iterative consultation process allowed unique perspectives of stakeholders to be captured and considered for NAS development.

#### 2.2.1 Development of the first NAS prototype

A team of psychiatrists, medical practitioners and researchers utilized the results of the first stakeholder consultation and cluster analysis to produce the first NAS prototype. To ensure the validity of the questionnaire, the development of NAS was guided by a list of questionnaires that were used in the data included in the cluster analysis and/or items relating to core symptoms of neurodevelopmental disorders. The list of questionnaires included (in alphabetical order): the Antisocial Process Screening Device (APSD) (37); Autism Diagnostic Observation Schedule, Second Edition (ADOS-2) (38); Autism Diagnostic Interview-Revised (ADI-R (39); Brief Symptom Inventory (BSI) (40); Conners' Parent Rating Scale—Revised (41); Child Behaviour Checklist (CBCL) (42); Children's Sleep Habits Questionnaire (CSHQ) (43); Griffith Empathy Measure (GEM) (44); Mullen Scales of Early Learning (MSEL) (45); Social Communication Questionnaire (SCQ) (46); Strengths and Difficulties Questionnaire (SDQ) (47); Short Sensory Profile, Second Edition (SSP) (48); Preschool Form and School-Age Form of Social Responsiveness Scale, 2nd Edition (SRS-2) (49); Vineland Adaptive Behaviour Scales (Second Edition; Vineland-II) (50); and Wechsler Intelligence Scale for Children, Fifth Edition (WISC-V) (51). Developmental regression questions were also informed by Furley and colleague's (52) review.

The NAS questionnaire prototype included a demographics section to capture socio-demographics information (age, gender), medications currently used, known physical and mental health conditions, and family medical history. The second part of the prototype encompassed a detailed assessment of twelve categories of NDD-related symptoms and abilities, including: (a) communication, (b) emotions, (c) social, (d) behavior (including hyperactivity, impulsivity, hostility, conduct issues, callousness, as well as emotional and behavioral regulations), (e) cognitive, (f) obsession and compulsion, (g) repetitive restricted behaviors, (h) tics, (i) sensory, (j) motor ability, (k) adaptive functioning, and (l) physical health. Screening questions were included in all but the physical health section, such that detailed questions were only displayed if a responder selected “yes” to any of the screening questions, indicating potential concerns in a particular symptom or ability category. The NAS questionnaire was also designed so that questions irrelevant to the responders' age and/or ability could be skipped. The NAS prototype can be provided upon request.

#### 2.2.2 Second stakeholder consultation

The draft NAS questionnaire generated from the above analysis was then provided for a second stakeholder consultation to understand whether the NAS prototype met the needs of the stakeholders and aspects that needed to be revised. The second consultation involved the same group of stakeholders evaluating the first NAS prototype via an online questionnaire (see Supplementary Table S1) to understand: (a) if the screening questions in the initial draft could effectively guide responders to fill out relevant sections only (*Yes/No question*); (b) if the questions were important and suitable for NDD assessments (*Yes/No questions,* followed by *open-ended* feedback if “No” was selected); healthcare professionals were also asked whether the order of the questions was appropriate (*open-ended question*), if the NAS questions could effectively capture relevant NDD symptoms (*Yes/No question*), and if they would recommend adding/removing any questions (*Yes/No question* and *open-ended question*); (c) if language, structure, and response format of the questionnaire were appropriate (*multiple selections*, *Yes/No questions, and/or open-ended questions)*; (d) (for parents or adults with NDDs) the ideal environmental settings for NDD assessment (*open-ended question*); (e) how important it is for NAS to detect NDD compared to obtaining a details profile of the person being assessed (*multiple selections*); and (f) how best to present the NAS results (*multiple selections*). Participants were given opportunities to provide suggestions and elaborate on their responses. The qualitative data was collected between June and October, 2023, via an online questionnaire hosted in REDCap electronic data capture tool hosted and managed by Research Technology Services (UNSW Sydney). Responses from 18 participants were collected, that is, all of the stakeholders who agreed to participate in the second consultation, and 78.26% of the initial pool of 23 stakeholders.

### 2.3 Data analyses

Quantitative data was analyzed using IBM Statistical Package for the Social Sciences (SPSS) and Microsoft Excel. Qualitative responses were coded independently by at least two of the three authors (TYW, SIA, and CLC) using NVivo 14 software. Each coder coded the responses to the questionnaires independently and grouped the codes into themes and sub-themes guided by the grounded theory method (53, 54). Any emerging new themes were compared against the existing themes, and either became their own new themes or were used to expand and/or modify existing themes. The themes and subthemes between the three coders were then compared. In case of discrepancies, codes and questionnaire responses were reviewed and refined, and new themes were formed through consensus.

## 3 Results

### 3.1 Quantitative feedback on the prototype

Quantitative data was collected from stakeholders (parents/caregivers of children with NDDs, adults with NDDs, health professionals or clinicians working with children with NDDs, and a representative of a disability service provider). In terms of NAS prototype content, stakeholders tended to agree that the prototype was relevant and appropriate. All stakeholders (*n* = 18) agreed that the sections in the NAS prototype are important for the assessment of a child with NDD. Furthermore, all health practitioners and service providers (*n* = 6) agreed that the NAS prototype effectively captured the type of symptoms a child with NDD(s) may have. A minority of participants (*n* = 5; 27.78% of all 18 participants) suggested there were items/sections in the draft that were not suitable, and provided further comments. In addition, two health practitioners/service providers suggested additional questions could be included in the prototype. Suggestions were summarized as qualitative feedback. Finally, while all adults with NDD(s) and parents/caregivers (*n* = 12) agreed that NAS was important or very important for detecting NDDs, 50% of health practitioners/service providers (*n* = 3) also thought that NAS was important or very important in detecting NDDs and comorbidities (as opposed to building an assessment profile). All qualitative suggestions will be discussed in the “Qualitative Feedback on the Prototype” section below.

In terms of the logistics of assessment, 83.33% of all 18 participants (*n* = 15) agreed that the screening questions provided enough information to allow subsequent irrelevant questions to be skipped. In addition, most participants endorsed a 4-point scale to respond to NAS questions (“None of the time”, “Some of the time”, “Most of the time”, and “Not applicable”), regardless of whether the questions were about the child's daily function (endorsing participants *n* = 9; 50%), caretakers' level of distress (endorsing participants *n* = 11; 61.11%), or how a child's symptoms impacts people around them (endorsing participants *n* = 11; 61.11%). Furthermore, participants reported that they preferred NAS assessment results to be reported using graphs with visual indicators of the normal range (*n* = 11; 61.11%).

### 3.2 Qualitative feedback on the prototype

Three themes and six sub-themes were identified from the second consultation. The main themes were: (a) suitable features of the NAS draft, (b) modification suggestions on the NAS questionnaire; and (c) suggestions on the administration of NAS. The themes and associated sub-themes are illustrated in Table 1 and are discussed below.

**Table 1:** Themes and sub-themes emerged during the second stakeholder consultation.

Themes	Sub-themes
1. Suitable features of the NAS draft	Not applicable
2. Modification suggestions on the NAS questionnaire	1. Comprehensive diagnostic content 2. Applicable questions 3. Readability and language of the questionnaire
3. Pragmatic administration of NAS	4. Flexible Administration 5. Administration contexts6. Practitioner attributes

#### 3.2.1 Theme 1: suitable features of the NAS draft

Adults with NDD(s), parents, health practitioners, identified positive attributes of NAS. Some participants commented that the NAS covered a comprehensive range of symptoms, and was useful as an assessment scale for disorders. For instance, an adult with NDD(s) mentioned that the scale “should be helpful for both getting a diagnosis, and possibly an assessment tool that could be used for say NDIS to allocated resources”. Note that NDIS, or National Disability Insurance Scheme in full, is an Australian scheme that provides financial support for disability interventions. It was also mentioned that the order of the questionnaire was appropriate, and the items were easily understandable.

#### 3.2.2 Theme 2: modification suggestions on the NAS questionnaire

Stakeholders suggested some adjustments to the NAS prototype, which include modifying the content of the questionnaire (*Sub-theme 1*), showing relevant questions only (*Sub-theme 2*), and improving the language, grammar, and presentation of the questionnaire (*Sub-theme 3*).

##### 3.2.2.1 Sub-theme 1: comprehensive diagnostic content

Participants suggested modifications to various aspects of the questionnaire prototype. Regarding the content of the questionnaire, it was suggested that we should fine-tune the child's diagnoses, family medical history, physical pain questions, as well as the “social communication” and “communication” domains. Suggestions to child's diagnosis questions include asking for more detailed information, especially: (a) allowing NAS responders to indicate disorder(s) being diagnosed prenatal; (b) providing a chance for NAS responders to disagree with a child's diagnosis; (c) asking about who made the reported diagnosis/diagnoses; and (d) allowing reported speculated but yet-to-be diagnosed disorder(s). For example, a disability service provider mentioned that “[w]ith conditions such as Down syndrome and some other syndromes which can now be detected prenatally, the question relating to age of onset/diagnosis may need to include a “prenatally detected” option”. (corresponding to point a). In regard to point (b), an adult with NDD(s) suggested “asking the parents if they agree with the child's diagnosis”. Concerning who diagnosed a child (point c), a health practitioner suggested that “do you need to ascertain if the diagnosis has been made by a health professional OR are you ok if the parent has made a diagnosis by Dr Google/internet?”. Finally, a disability service provider raised the question “I wonder if there's value in adding an option of “suspected”?” (point d). It is worth noting that parents did not provide suggestions on the child's diagnostic questions.

Suggestions were also made on modifying the family medical history, physical pain questions, and the “social communication” and “communication” domains. Regarding family medical history, it was suggested that cousins' conditions should also be taken into consideration, and that “other” and “unknown” options should be provided. It was also suggested that the question “level of the impact of [a family member's disorder] on the functioning of the child's [diagnosis]” should be reworded as an adult with NDD(s) suggested that he/she “was confused by the wording of the questions.” Note that no health professionals or parents provided feedback on the family medical history questions. Aside from family history questions, an adult with NDD(s) suggested that physical symptoms should also be assessed, in case “if a person's NDD is physical this is something that could have a significant impact upon both their functioning and their wellbeing”. Regarding the “social communication” and “communication” domains, an adult with NDD(s) suggested that for the social communication domain, “there should be a question about if the child can maintain long[-]term friendships”. Another health practitioner suggested that the “communication” domain should be expanded to cover a wider spectrum of language difficulties, “e.g., speech sound disorder and fluency problems”.

Furthermore, adults with NDDs, health practitioners, and parents suggested that NAS respondents should be allowed to enter free text and to elaborate on their responses, and provided with further information for some questions. For example, regarding free text, a health practitioner said “I have selected an open-ended response in the above [all categories of] questions as I think it allows more space for people to describe what felt important to them”. However, the same health practitioner also noted that they were “aware that it might not be suitable for every parent or caregiver”. Similarly, regarding questions related to a child's feelings or emotions, an adult with NDD(s) suggested providing an option for parents/caretakers to flag that the emotion reported was observed by the parent/caretaker instead of being expressed by the child being assessed. In addition, adult with NDD(s) and health practitioner participants suggested that examples should be given to explain the questions better, for example, a health practitioner said, “I wonder whether examples might be provided”.

In addition, it was recommended that the strengths and all aspects of challenges faced by children with NDDs should be considered. Regarding strengths, one adult with NDD(s) said “[y]ou could include a mention to child's strengths and talents”. Similarly, an adult with NDD(s) also suggested taking a child's academic excellence into account: “[in the] cognitive domain an option for parents to express that their child excels academically may be useful”. Concerning challenges, an adult with NDD(s) suggested that NAS has to “capture the spectrum of different challenges people may be experiencing … as well as things they find tricky”.

There are some other suggestions related to the content of NAS in general. For example, when assessing family medical history, we should be mindful that formal diagnoses of some disorders might not have previously existed. Furthermore, we should be aware that some disorders are not well-understood by the general public (e.g., mood issues and oppositional defiant disorder), symptoms of some disorders are hard to distinguish (e.g., ASD and obsessive-compulsive disorder), and some disorders are traditionally diagnosed by a pediatrician (e.g., global developmental delay and ASD). A health practitioner also reminded that we should reconsider the rationale of having screening questions. On the note of comorbidities, it was suggested that an algorithm system could be employed to identify the primary diagnosis and to assess comorbidities if ASD was identified. A health practitioner also proposed that if the goal of NAS was to collect all symptom information, then formal diagnoses may not need to be reported in NAS. Finally, regarding tics and Tourette's Syndrome, it was suggested that enquiries about the improvement of the condition is irrelevant due to the fluctuating nature of the symptoms.

##### 3.2.2.2 Sub-theme 2: applicable questions

Participants suggested that is it important to only show relevant questions to NAS users. One adult with NDD(s) suggested that “I think the branching logic is great and very important … as not all questions will be relevant for each individual”. A health practitioner also pointed out that NAS could be revised to only show age-appropriate questions, as questions such as “is your child able to stop and think before acting?” and “does your child experience intense emotions that are difficult to control?” might not be useful in identifying symptoms in younger children. Note that no parents provided comments on this topic.

##### 3.2.2.3 Sub-theme 3: readability and language of the questionnaire

There were suggestions on the language, grammar, and presentation of the questionnaire prototype. Some participants commented that the domain titles were inappropriate. For example, an adult with NDD(s) said, “[m]ight be a bit confronting and unhelpful to be reading this when you are just wanting people to answer based on what their child's experience is (regardless of what labels they have been exposed to)”. Another health practitioner mentioned “[t]he heading for the sub-sections might also create response bias to the respondents”, which echoed the comment from an adult with NDD(s): “I wonder whether the inclusion of domain and subdomain headings on the final version of the questionnaire will influence people's responses?”. Nevertheless, parents did not raise any concerns about the domain titles.

All three groups of participants also raised suggestions on the word choices of the NAS prototype. Some words that were suggested to be replaced included “*overly*/*excessively*” (because they have negative connotations), “*normal*” (an adult with NDDs mentioned that “if the NAS is filled out by a parent who is used to their child's behaviours … their answers may be slightly skewed because they may view those behaviours as “normal” for that child”), “*acceptable*” (what is regarded as acceptable can be subjective), and “*disease*”. It was suggested that the choice of words in the social domain was too arbitrary at times and should be revised. Furthermore, all three groups of participants raised concerns regarding the use of phrases “*age of onset*” and “*trigger*” in the child's health history questions, as (a) these phrases could lead to confusion, such as “is this referring to age of diagnosis, or age of when symptoms first started being noticed?” (comment from an adult with NDDs), and (b) that some disorders are congenital and therefore do not have a time of onset or triggers.

In terms of readability, participants suggested that the language of the questionnaire was too difficult at times, and some minor grammatical mistakes should be fixed. For instance, a parent reported that “I would say across the entire survey the language level is quite high and most people who need to complete this would require a higher level of literacy”. A health practitioner commented “I am thinking whether items under cognitive domains might be a bit challenging for parents to understand just by reading”. Similarly, an adult with NDD(s) suggested titles such as “interpersonal sensitivity” and “affective empathy” should be written in lay language. To improve the readability, it was suggested the team invite individuals with NDD to review the questions, and/or have an easy-to-read version for NAS. In addition to readability, it was suggested that the team go through the NAS questionnaire and correct grammatical errors.

All three participant groups also provided feedback on the format of NAS to enhance its accessibility. Suggestions include (a) adding clear instructions of what the next question would be based on the current response; (b) using headings, different font sizes and colors to distinguish between sections; (c) re-ordering questions to present sections that are more easily understood first (e.g., physical health); and (d) consider using a 10-point scale for responses (cf. the quantitative findings under “Quantitative Feedback on the Prototype”).

Finally, participants from all three groups were concerned about the length of the questionnaire. Nevertheless, some parent and health practitioner participants thought the length was acceptable to cover the complex NDD presentations. To shorten the questionnaire, participants recommended grouping or truncating questions on the child's and family's mental health and medical history.

#### 3.2.3 Theme 3: pragmatic administration of NAS

Participants also provided suggestions on how NAS should be administered. Specifically, participants suggested that NAS administration should be flexible (*Sub-theme 4*). They also provided opinions on assessment contexts (*Sub-theme 5*) and practitioner attributes (*Sub-theme 6*).

##### 3.2.3.1 Sub-theme 4: flexible administration

Participants recommended providing extra information or support whilst individuals fill in the NAS questionnaire, with one such example being the purpose of the questionnaire. An adult with NDD(s) suggested that “[it] would also really help with increasing understanding of why people should complete [NAS]”. Similarly, it was suggested that the purpose of the NAS results should be better explained to participants. In addition, participants also suggested providing extra resources or support whilst individuals fill in the NAS questionnaire. For instance, support should be provided to ensure that the questionnaire respondents understand the NAS questions. A health practitioner commented that “I wonder if there could be ways to make sure the questions are understood in the ways that they are intended to. I think parents might need help when they are filling in the survey”. It was also suggested that support or information should also be given if NAS responders find certain questions upsetting or confronting.

Further to providing information and support, participants also advised NAS administrators to consider individual needs and ways to accommodate them accordingly when administering NAS. Special considerations included: (a) preparing multiple versions of NAS to accommodate a child's age and the responders' physical ability/accessibility requirements (e.g., providing different delivery formats including an audio version/screen reader, allowing a person to respond to NAS verbally or physically using a computer or on paper, and providing appropriate font size), (b) allowing assessments to be done across multiple sessions and providing extra response time if needed, (c) allowing assessment without children and/or provide an assessment location friendly to children, and (d) measures (e.g., inform about the assessment progress) should be put in place to keep interviewees engaged).

##### 3.2.3.2 Sub-theme 5: administration contexts

Participants were also concerned about the assessment location. Participants suggested that the NAS assessment be done in a comfortable location. For example, an adult with NDD(s) and a parent suggested that NAS should be administered in a familiar location/environment. The assessment environment should also be flexible according to the characteristics of the child, and should be comfortable for the assessment scale responder. Other suggested environmental features include having low light, no clock, and being in a “not-crowded” setting. Some responders suggested that assessments should be done face-to-face, and suggested locations such as clinics, home, and schools. However, a parent mentioned that an online or telehealth option should be provided for individuals to suit individual needs, for example “[O]nline/digital appointments would be a great idea for many with TS [i.e., Tourette syndrome]”.

##### 3.2.3.3 Sub-theme 6: practitioner attributes

Participants also discussed some practitioner attributes that they would appreciate. Adults with NDD(s) suggested that they preferred the assessment to be administered by a health practitioner that they are familiar with. One of the participants explained that this was because “[i]f an older child is self-completing needs support to answer questions, it would be important that this support person knows them well and is familiar with their communication style”. Some other important attributes included that the assessment atmosphere should be welcoming, and the experience with the clinician should be pleasant.

## 4 Discussion

The current study aimed to describe the co-design and developmental process of NAS, with a focus on obtaining stakeholder feedback on the first NAS prototype. The prototype covered symptoms in the areas of communication, emotions, social, behavior (including hyperactivity, impulsivity, hostility, conduct issues, callousness, as well as emotional and behavioral regulations), cognitive, obsession and compulsion, repetitive restricted behaviors, tics, sensory, motor ability, adaptive functioning, and physical health. Comments from stakeholders suggested that while NAS would be useful, further adjustments were necessary to improve the content, assessment flow (only show relevant questions), the language/clarity, and the presentation of the prototype. Suggestions were also made on how NAS should be administered. It was advised that the assessment arrangement and location should be flexible, and the practitioner should be welcoming and familiar to the person being assessed. NAS was then modified according to stakeholders' feedback.

The results of the study highlighted the need to develop a transdiagnostic neurodevelopmental disorder assessment scale. This was evidenced not only by participants' feedback on NAS being comprehensive and potentially useful, but also from the modification suggestions. Specifically, there were suggestions of including extra symptoms and information, which underscored the fact that a wide range of presentations should be considered while assessing a child with NDDs. This is consistent with the findings that individuals with a primary NDD diagnosis could show symptoms of other NDD diagnoses, even those symptoms were not part of the diagnostic criteria of the primary NDD diagnosis (55). In fact, symptoms of NDDs evolve across the developmental span of individuals (56, 57). Specifically, some symptom constellations may take prominence and meet the diagnostic criteria, while others may remain sub-threshold at a given time-point (58, 59). In addition, it was found that symptoms “irrelevant” to a primary diagnosis could nonetheless predict the primary disorder being diagnosed in the future (e.g., early motor development delay associated with future ADHD diagnosis) (60). The symptom manifestation pattern highlights the fact that NDD assessment should be transdiagnostic and should capably consider a board range of symptoms, in order to provide necessary support, timely discovery of comorbidities, and prevent symptoms from developing into severe psychopathology.

### 4.1 Strengths, limitations, and future directions

A strength of the current study is that stakeholders were substantially involved in co-designing NAS. Co-design can enhance the developmental process of a health problem by providing new insight into the problem and possible solutions (36). The benefits are also observed in the current project. For example, stakeholders provided comments on what information should be gathered by NAS, how to adjust the language to make it more acceptable to future end-users, and how to streamline the NAS assessment process. Comments from stakeholders ensure the rigor of the NAS scale and allow it to be both efficient and useful to individuals with NDDs, as a career and/or diagnosis makers. Overall, the study results underscore the importance of involving comments from stakeholders, as stakeholders provided unique insight from their experience as end-users.

The current study design has several limitations. Due to recruitment difficulties, some stakeholders were lost between the two stakeholder consultations of the study. Although we successfully gathered comments from 18 participants in the second consultation, which is an acceptable number (61, 62), the loss of information from attrition may bias the information received. Moreover, stakeholders' socioeconomic background was not taken into account in the current study. Future studies should investigate how NAS could be adapted to suit the needs of individuals from different socioeconomic backgrounds. In addition, while NAS prototype has face validity after the rigorous development process by stakeholders and researchers, its psychometrics properties remain unknown. Future work will be conducted to investigate its validity, reliability, and real-world applicability. Furthermore, future work will continue with this theme of co-design upon completion of the NAS scale to allow for further fine-tuning of NAS administration.

## 5 Conclusion

The current article described the co-design process used in the development of NAS, a transdiagnostic NDD assessment, with a focus on the stakeholder feedback on the first NAS prototype. The results presented underscore the importance of having a transdiagnostic NDD assessment that covers a wide range of symptoms, and suggestions for further modifications to be made to improve the content, assessment flow, language, and presentation of NAS. NAS prototype will be amended according to stakeholders' feedback, and the validity and reliability of NAS will be investigated in future work.

## Data Availability

The raw data supporting the conclusions of this article will be made available by the authors, without undue reservation.
