# Supplementary material for: Co-design of the neurodevelopment assessment scale
Source: Front Child Adolesc Psychiatry. 2025 Jan 28;4:1497632. doi: 10.3389/frcha.2025.1497632 (PMC11811075; doi:10.3389/frcha.2025.1497632)
Supplement: Supplementary file 1 [file Table1.pdf]

Supplementary Material

Supplementary Table 1

List of Questions for the Second Consultation

**Questions for parents of children with neurodevelopmental disorders and adults with neurodevelopmental disorders**	**Questions for health practitioners and disability service providers**
Do the initial screening questions provide enough information to determine which domains could be skipped in the assessment of a child who does not demonstrate abilities in that area based on the results of the initial screening question?<br>Yes / No	
Are the sections included in this draft Neurodevelopmental Assessment Scale (NAS) important for the assessment of a child with a Neurodevelopmental Disorder (NDD)?<br>Yes / No<br>If yes, please provide further details:	
(No corresponding question)	Do you have any comments on the order of the sections in this draft version of the NAS?
Are there any items/sections in this draft NAS that are not suitable?<br>Yes / No<br>If yes, please provide further details:	
Please list any parts/sections of the draft NAS that might not be understandable or accessible to someone with an NDD? Do you have any suggestions to rephrase/improve them?	
(No corresponding question)	In your experience, are there any additional items/questions that you think should be included, that were not included in the current draft? (If the current sections cover the most important topics, you can move on to the next section.)<br>Yes / No<br>If yes, please list:
(No corresponding question)	Please list any sections/questions you would remove to make the NAS more concise?
Do you have any comments on the overall format or structure of the NAS?<br>Yes / No<br>If yes, please provide further details:	
(No corresponding question)	Do you think the draft NAS effectively captures the type of symptoms a child with

<table>
<tr><th>Questions for parents of children with neurodevelopmental disorders and adults with neurodevelopmental disorders</th><th>Questions for health practitioners and disability service providers</th></tr>
<tr><td></td><td>an NDD may have?
Yes / No</td></tr>
<tr><td colspan="2">We aim to develop a NAS which captures each family/child's specific goals and priorities. In your opinion, what would be the best way to capture the impact of each item/section on 1) daily functioning, 2) level of distress, or 3) other aspects of the child's life?
Please select your preferred question(s), and drop-down response options to capture the impact of NDDs:
1. To what extent does this impact your/their daily life?
2. To what extent does this cause you/them distress?
3. To what extent does this impact the parent's/carers/significant others?

Possible responses (each participant could only choose one option):
1. Four options: "None of the time", "Some of the time", "Most of the time", "Not applicable"
2. Five options: (1) Strongly disagree, (2) Disagree, (3) Neither agree nor disagree, (4) Agree, (5) Strongly agree
3. Three Options: "Usually", "Sometimes", "Never"
4. Scale from 0-100% (with anchor points adapted to the context of each question)
5. Score: 0,1,2,3,4,5
6. "True", "False"
7. Descriptive options, for example when asking about "unusual eye contact", there could be two options: a. Appropriate gaze with subtle changes mixed with other communications; b. Use poorly modulated eye contact to initiate, terminate, or regulate social interaction
8. Open-ended response
9. Other, Please provide further details: _______________</td></tr>
<tr><td>We understand that the setting in which the assessment takes place is important in allowing the child to demonstrate their strengths. Please list any advice you would give to clinicians in preparing a suitable environment to administer the NAS (for example face to face, digital/online, phone etc.)?</td><td>(No corresponding question)</td></tr>
<tr><td colspan="2">In your opinion, what is the most useful way for assessment results to be presented? Select all that apply:
1. Visually in a table
2. Visually in a graph with a "typical" range shaded to show where the results lie
3. One summary paragraph
4. Key dot points for each section</td></tr>
<tr><td>Overall, in your opinion how important is the draft NAS for detecting NDDs in</td><td>Overall, in your opinion how important is the draft NAS for detecting NDDs</td></tr>
</table>

**Questions for parents of children with neurodevelopmental disorders and adults with neurodevelopmental disorders**	**Questions for health practitioners and disability service providers**
childhood?<br><br>Possible responses (each participant could only choose one option):<br>1. Not important<br>2. A little important<br>3. Somewhat important<br>4. Don't know<br>5. Important<br>6. Very important	(including comorbidities) versus ascertaining the detailed profile?<br><br>Possible responses (each participant could only choose one option):<br>1. Not important<br>2. A little important<br>3. Somewhat important<br>4. Don't know<br>5. Important<br>6. Very important
Do you have any comments you wish to add?<br>Yes / No<br>If yes, please comment below: _______________	
